# Mutation of DNA Polymerase β R137Q Results in Retarded Embryo Development Due to Impaired DNA Base Excision Repair in Mice

**DOI:** 10.1038/srep28614

**Published:** 2016-06-30

**Authors:** Feiyan Pan, Jing Zhao, Ting Zhou, Zhihui Kuang, Huifang Dai, Huan Wu, Hongfang Sun, Xiaolong Zhou, Xuping Wu, Zhigang Hu, Lingfeng He, Binghui Shen, Zhigang Guo

**Affiliations:** 1 Jiangsu Key Laboratory for Molecular and Medical Biotechnology, College of Life Sciences, Nanjing Normal University, 1 WenYuan Road, Nanjing, 210023, China; 2 The Second Hospital of Nanjing, The Second Affiliated Hospital of Southeast University, 1-1 Zhongfu Road, Nanjing, 210003, China; 3 Departments of Radiation Biology and Molecular Medicine, City of Hope National Medical Center and Beckman Research Institute, Duarte, CA, 91010, USA

## Abstract

DNA polymerase β (Pol β), a key enzyme in the DNA base excision repair (BER) pathway, is pivotal in maintaining the integrity and stability of genomes. One Pol β mutation that has been identified in tumors, R137Q (arginine to glutamine substitution), has been shown to lower polymerase activity, and impair its DNA repair capacity. However, the exact functional deficiency associated with this polymorphism in living organisms is still unknown. Here, we constructed Pol β R137Q knock-in mice, and found that homozygous knock-in mouse embryos were typically small in size and had a high mortality rate (21%). These embryonic abnormalities were caused by slow cell proliferation and increased apoptosis. In R137Q knock-in mouse embryos, the BER efficiency was severely impaired, which subsequently resulted in double-strand breaks (DSBs) and chromosomal aberrations. Furthermore, R137Q mouse embryo fibroblasts (MEFs) were more sensitive to DNA-damaging reagents, such as methyl methanesulfonate (MMS) and H_2_O_2_. They displayed a higher percentage of DSBs, and were more likely to undergo apoptosis. Our results indicate that R137 is a key amino acid site that is essential for proper Pol β functioning in maintaining genomic stability and embryo development.

The genome is constantly being damaged by a variety of endogenous and exogenous DNA-damaging agents. To repair these damages, cells have developed several DNA repair mechanisms, which are essential for maintaining the integrity of genomic DNA. Defects in DNA repair systems lead to a failure in faithful repair, which can cause genetic mutations and can subsequently contribute to genomic instability and the initiation of cancer^1^. Base excision repair (BER), one of the main repair pathways in eukaryotic cells, fixes DNA base damage caused by endogenous and exogenous reagents^2^,^3^. BER is initiated by the excision of the damaged base by a specific DNA glycosylase, followed by the incision of the DNA backbone by AP endonuclease 1 (APE1) to produce a nicked abasic intermediate^4^. This intermediate structure can be processed through either the short patch BER (SP-BER) or the long patch BER (LP-BER) pathway^5^,^6^. In the former process, DNA Pol β adds only one nucleotide to the 3′-end of the nicked AP site, and then the dRP lyase activity of Pol β catalyzes β-elimination of the 5′-sugar phosphate residue, resulting in a ligatable nick which can then be sealed by XRCC1/Ligase IIIα^7^,^8^. In the latter process, Pol β or the alternative Pol δ performs strand displacement synthesis, generating a short, 2–10 nt DNA flap, which is removed by flap endonuclease 1 (FEN1)^9^,^10^,^11^,^12^,^13^. DNA ligase I then seals the nick^5^.

Pol β, a 39 kDa protein, has been demonstrated to be a key player in both the SP-BER and LP-BER pathways^13^,^14^,^15^. Pol β contains two domains, a dRP lyase domain (8 kDa) and a polymerase domain (31 kDa). The dRP domain possesses dRP lyase activity, which acts to remove the sugar phosphate group, while the polymerase domain is responsible for the incorporation of new deoxyribonucleotides^16^. In addition, many proteins, including APE1, PCNA, and FEN1, are reported to interact with Pol β^17^,^18^,^19^,^20^. These protein-protein interactions are very important for the BER process, as they can recruit downstream factors to the DNA repair site, reciprocally stimulate enzyme activities, and coordinate the highly ordered chemical reactions involved in BER. Pol β deficiencies have been shown to impair BER efficiency, and to cause cells to be hypersensitive to alkylating or oxidative agents^12^,^21^. The knockout of Pol β in mice abolishes BER and causes the mutant cells to be hypersensitive to DNA damaging reagents, such as methyl methanesulfonate (MMS) and H_2_O_2_, which can result in early embryonic lethality. Pol β mutations that impair its polymerase activity or its interaction with other proteins have been shown to result in defective BER *in vitro*^22^,^23^,^24^,^25^. Mouse models encoding Pol β with reduced DNA polymerase activity develop an autoimmune pathology^26^. The knock-in of Pol β mutations in mice also impairs BER, and causes low birth rates and abnormalities embryo development^27^.

Pol β mutations have been detected in most types of human cancers^28^. These findings suggest that Pol β variants may result in biochemical alternations, BER deficiencies, and a predisposition to cancers^22^,^29^,^30^,^31^,^32^. Findings from studies of mouse models with the Y265C Pol β variant from the Sweasy laboratory also indicate that Pol β mutations may impair BER and lead to developmental delays and autoimmune diseases^26^,^27^. However, whether there are other Pol β mutations that increase the susceptibility to cancer, immune diseases and developmental abnormalities has not been thoroughly studied. It has been reported that the Pol β R137Q variant has a significantly reduced polymerase activity and impaired BER efficiency, which subsequently contributes to genomic instability and the development of cancer^20^. Here, we constructed Pol β R137Q knock-in mice to investigate whether the mice carrying this variant had any phenotypes indicative of these deficiencies. Our *in vivo* data show that the knock-in mouse embryos were small and had an increased mortality rate. This suggests Pol β R137Q polymorphism may impair the BER efficiency, which might subsequently contribute to genomic instability and developmental abnormalities.

## Results

### Generation of knock-in mouse model

**Figure 1:**
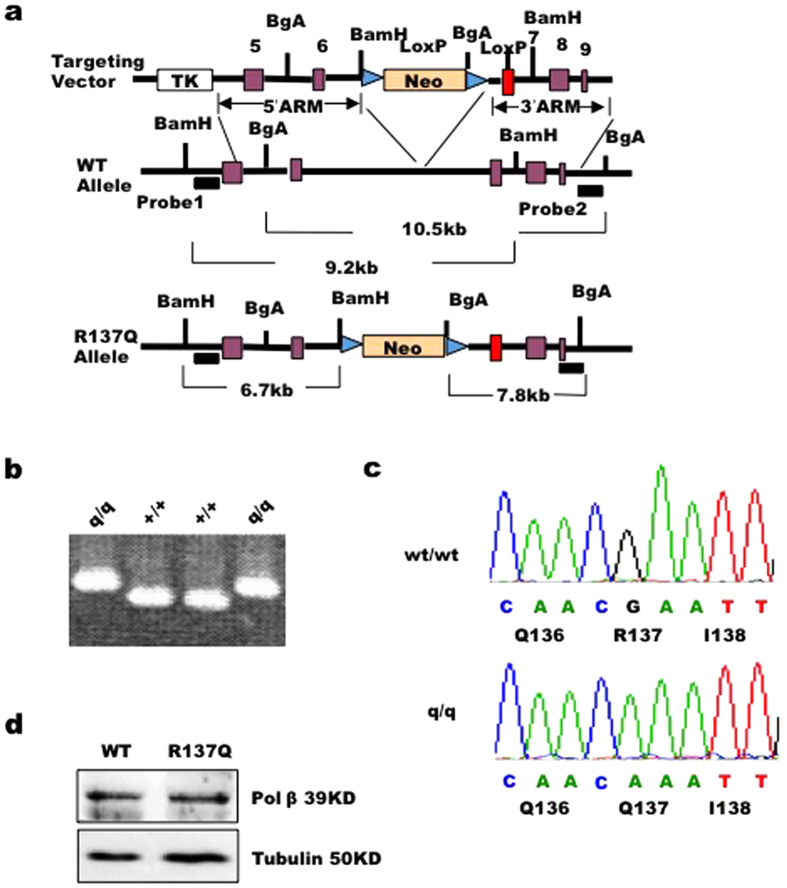
Generation of R137Q knock-in mice. (**a**) Partial restriction map of the R137Q targeting vector, the mouse *pol β* genomic locus, and the structure of the locus after combination. The targeting construct contains 5′-exon 5–6 and 3′-exons 8–9. Homologous recombination within the genomic sequence introduces the neo gene and the R137Q mutant exon 7. Restriction endonuclease sites used for cloning are shown in the panel. (**b**) To confirm that the mutation was inserted correctly, total RNA was extracted and Pol β cDNA was amplified via PCR. The unique EcoRI site within the DNA fragment was abolished by the targeted insertion of the mutant and thus confirmed the targeting. (**c**) The DNA sequence encoding the 137 amino acid residue of Pol β was confirmed via direct DNA sequencing. (**d**) Western blot analysis indicates that WT and R137Q mice have the similar expression levels of Pol β.

To examine the exact effects of the Pol β R137Q mutation in living organisms, we successfully constructed Pol β R137Q knock-in mice using the traditional gene targeting methods described in a previous study^33^ (Fig. 1a). The genotypes of the knock-in mice were confirmed via PCR assay and DNA sequencing (Fig. 1b,c). A Western blot assay showed that WT and R137Q mice possessed similar levels of Pol β expression (Fig. 1d).

### Homozygous Pol β R137Q mouse embryos are small

**Figure 2:**
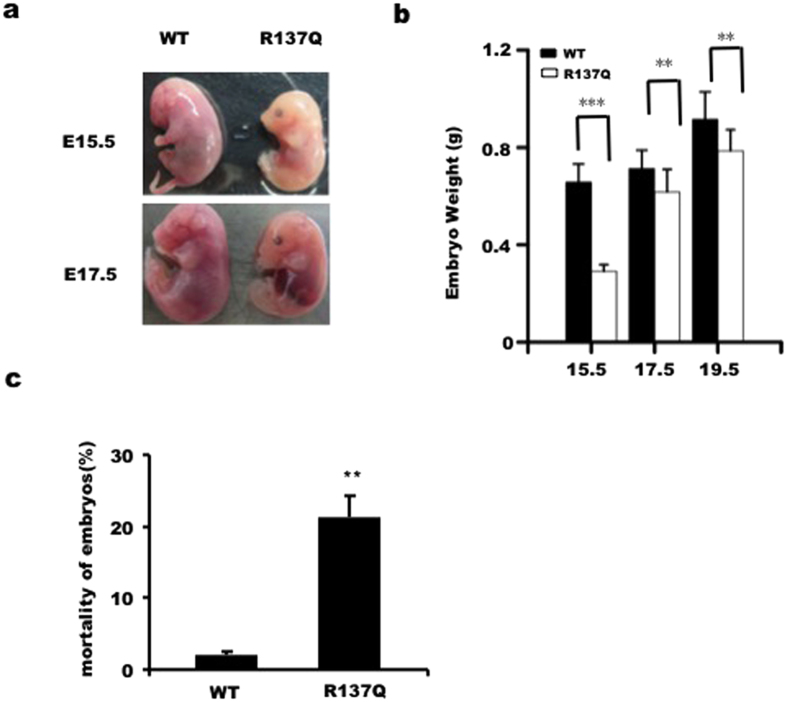
The R137Q mutation results in small embryos and embryonic lethality. (**a**) Pol β R137Q embryos were found to be smaller than WT embryos at E15.5 and E17.5. In addition, some R137Q embryos were dead. (**b**) The average embryo weights at E15.5, E17.5 and E19.5 show that R137Q embryos developed at a slower rate compared to WT embryos. The data represent the mean ± SD of seven to ten embryos (**P < 0.01, ***P < 0.001, Student’s t-test). (**c**) Approximately 21% of R137Q embryos were dead. The data represent the mean ± SD of embryos from eight pregnant mice (embryo numbers: WT = 54, R137Q = 41) (**P < 0.01, Student’s t-test).

After the knock-in mice were verified to be successfully expressing the mutated gene, they were bred normally. However, we found that the number of animals per litter in the R137Q group was significantly less than that in the WT group. To further investigate the underlying causes of this, we dissected the embryos from timed pregnant R137Q knock-in mice at specific times. We found that the homozygous R137Q mouse embryos at E15.5 d and E17.5 d were much smaller than the WT embryos (Fig. 2a). Body weight of embryos was also significantly reduced in R137Q mice (Fig. 2b). Moreover, dead embryos frequently observed while dissecting pregnant females. The morality of WT and R137Q embryos was 2% and 21%, respectively, based on an analysis of 54 WT embryos and 41 R137Q embryos (Fig. 2c). These results show that the process of embryo development is impaired by the Pol β R137Q mutation.

### R137Q mouse embryos exhibit reduced cell proliferation and increased cell apoptosis

**Figure 3:**
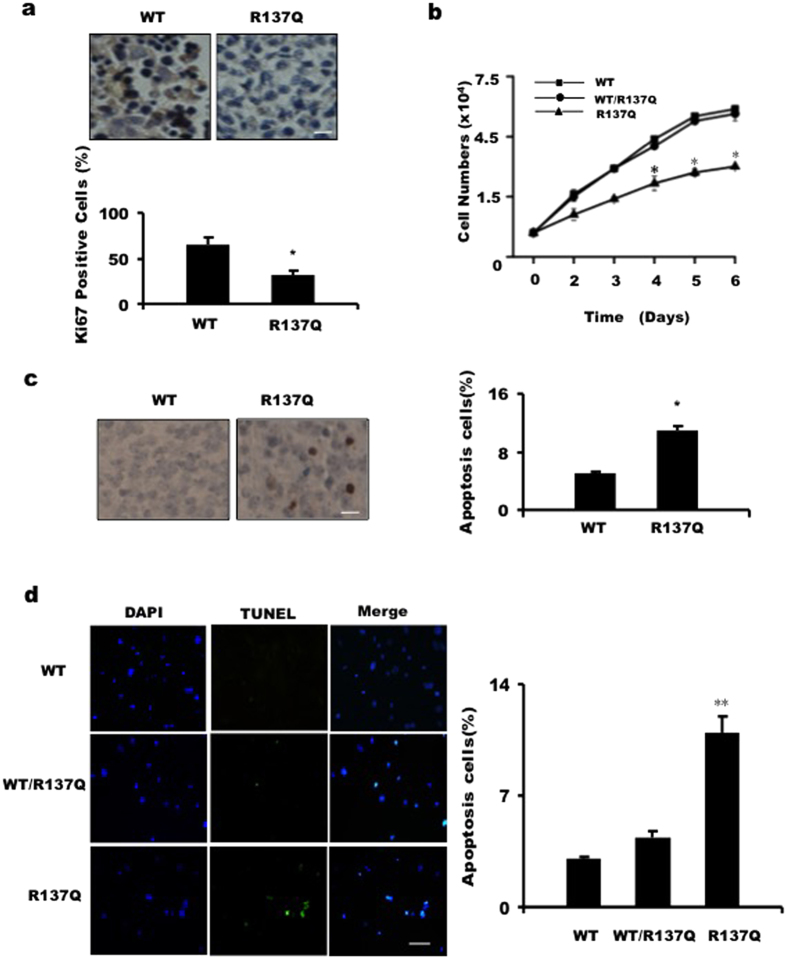
R137Q knock-in mouse embryos have lower levels of cell proliferation and higher levels of cell apoptosis. (**a**) Immunohistochemical sections (40×) of E16.5 embryos from R137Q mice showed less Ki67 positive cells. Scale bar: 20 μm. Cells (3 × 10^3^) were analyzed, and the percentages of positive cells (dark) were calculated and plotted. The data represent the mean ± SD from three independent experiments. (*P < 0.05, Student’s t-test). (**b**) We measured MEF proliferation rates using continuous cell counting for 6 days. Proliferation of R137Q MEFs was significantly slower than that of WT and WT/R137Q MEFs. The data represent the mean ± SD from triplicate wells (*P < 0.05, Student’s t-test). This experiment was repeated three times. (**c**) TUNEL analysis was applied to detect apoptosis. More TUNEL-positive cells (dark brown) appeared in sections from R137Q embryos. Cells (3 × 10^3^) were analyzed, and the percentages of positive cells were calculated and plotted. The data represent the mean ± SD of three independent experiments (*P < 0.05, Student’s t-test). Scale bar: 20 μm. (**d**) A stronger TUNEL-positive fluorescence signals (green color) was detected in R137Q MEFs. Scale bar: 50 μm. Cells (3 × 10^3^) were examined at 200× magnification. Percentages of positive cells were calculated and plotted. The data represent the mean ± SD of three independent experiments (**P < 0.01, Student’s t-test).

To understand the mechanisms responsible for the reduced size of R137Q embryos, we used Ki67 to characterize cellular proliferation in the tissues of the embryos and found that there was less Ki67 localization to the tissues from R137Q mice (Fig. 3a). To confirm this result, we cultured the mouse embryonic fibroblasts (MEFs) *in vitro* and constructed cell growth curves based on cell counts. As shown in Fig. 3b, R137Q MEFs proliferated slowly when compared with WT MEFs. These data indicate that decreased cell proliferation may contribute to the small size of R137Q embryos. Next, a terminal deoxytransferase-mediated dUTP biotin nick-end labeling (TUNEL) assay was used to examine cell apoptosis. As shown in Fig. 3c, the ratio of apoptosis was nearly 2-fold greater in R137Q embryos than in the WT group. Moreover, MEFs were used to measure cell apoptosis *in vitro*. The fluorescent images show that a greater signal was detected in the R137Q MEFs, which indicates that R137Q MEFs undergo higher levels of apoptosis than do WT or WT/R137Q MEFs (Fig. 3d). Collectively, these results suggest that increased apoptosis may also be responsible for the small size of R137Q embryos.

### R137Q reduced the base excision repair efficiency

**Figure 4:**
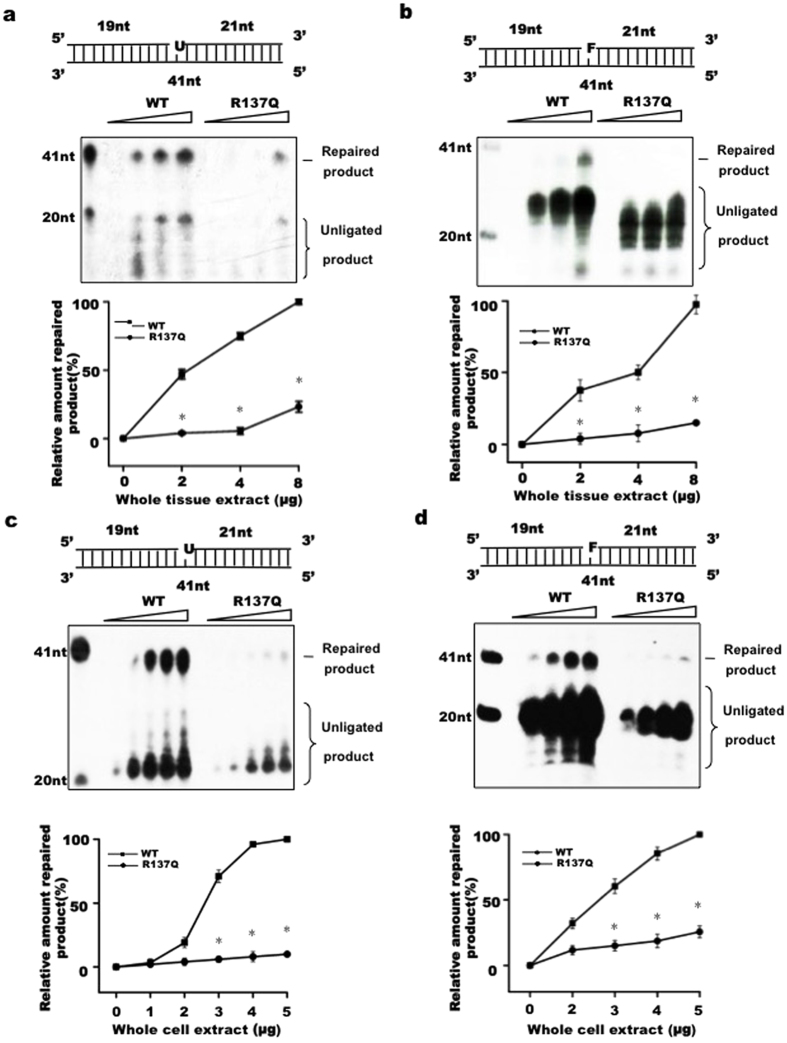
The R137Q homozygous embryos have reduced BER efficiencies. WT and R137Q tissue extracts were used in tests of SP-BER with the substrate pol-U (**a**) and LP-BER with the substrate pol-F (**b**). WT and R137Q MEF lysates were used in the SP-BER (**c**) and LP-BER (**d**) measurements. The top part of each panel shows the schematic structure of the corresponding DNA substrates. The middle portion shows the PAGE-separated products, and the bottom portion shows the relative percentage of repaired products at different enzyme concentrations, as indicated. Values represent the mean ± SD of six independent assays (*P < 0.05, Student’s t-test).

As Pol β plays important roles in the DNA repair process, and Pol β R137Q variants have already been reported to possess reduced polymerase activity. We therefore hypothesized that the impaired biochemical functions of the R137Q variant could result in reduced cell proliferation and high rates of apoptosis. To test this hypothesis, we examined the functional efficiency of Pol β R137Q in base excision repair. Equal amounts of tissue extracts from WT and R137Q mouse embryos were prepared to examine BER efficiency (see Supplementary Fig. S1). Uracil-containing substrate (Pol β-U) and tetrahydrofuran-containing substrate (Pol β-F) were used for the SP- and LP-BER assays, respectively. The incorporation of ^32^P-dCTP and other deoxynucleotides produced 20–30 nt non-ligated intermediates, and fully repaired 41 nt products were detectable using a phosphorimager. We observed that the uracil or THF lesions were efficiently repaired in the presence of 8 μg of whole tissue extract from WT mice, whereas the repair efficiency by the same volume of R137Q mutant tissue extract was only approximately 30% and 20% for SP- and LP-BER, respectively (Fig. 4a,b). The significant differences between the WT and R137Q samples in terms of SP-/LP- BER efficiency were further confirmed using MEF extracts (Fig. 4c,d). In addition, our data showed that for the same extracts, LP-BER efficiency was much lower than SP-BER efficiency. This difference may be due to the different activities of Ligase I and Ligase IIIα, which catalyze the LP-BER and SP-BER reactions, respectively.

### More DSBs and chromosomal aberrations found in R137Q embryos and MEFs

**Figure 5:**
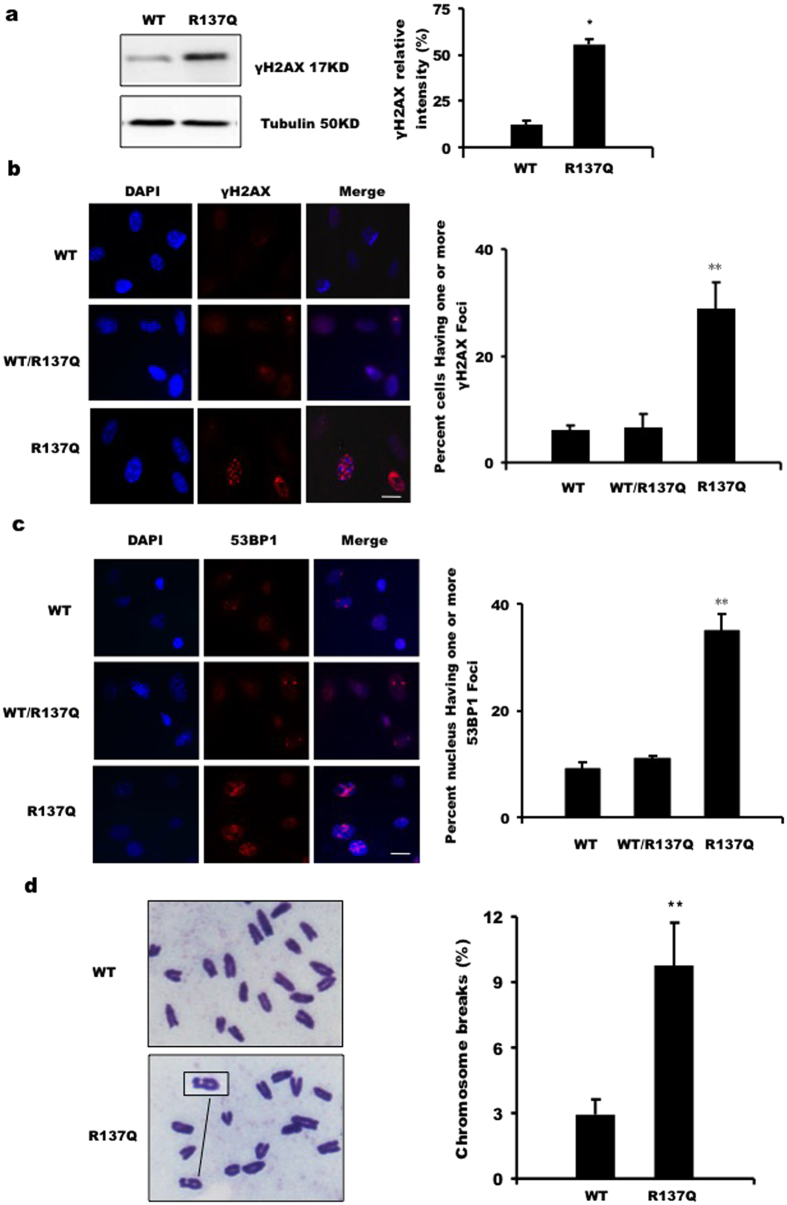
Tissues and MEFs from R137Q embryos show DSBs accumulation and increasing levels of chromosome breaks. (**a**) The protein levels of γH2AX in tissues from WT and R137Q embryos were determined via Western blot analysis. γH2AX bands intensities obtained via quantitation from a fluorimager analysis, with results normalized to Tubulin. The data represent the mean ± SD of three independent experiments. *P < 0.05, Student’s t-test. (**b**) The γH2AX foci in WT, WT/R137Q and R137Q MEFs were detected via immunofluorescence analysis as previously described. Fifty random regions were examined at 400× magnification. Nuclei containing ≥1 foci (red) were counted as positive for γH2AX foci formation. Cells (3 × 10^3^) were analyzed and the percentage of positive cells were calculated and plotted. The data represent the mean ± SD of three independent experiments. **P < 0.01, Student’s t-test. Scale bar: 10 μm. (**c**) The 53BP1 foci in WT, WT/R137Q and R137Q MEFs were also examined via immunofluorescence analysis. Nuclei containing ≥1 foci (red color) were counted as positive for 53BP1 foci formation. Cells (3 × 10^3^) were analyzed and the percentages of positive cells were calculated and plotted. The data represent the mean ± SD of three independent experiments. **P < 0.01, Student’s t-test. Scale bar: 10 μm. (**d**) After colcemid treatment (0.1 μM, 5 h), cells were harvested and stained with a Giemsa solution, examined under a microscope (100×) for mitotic cells and imaged using NIS-Elements D 3.0. The chromosome number of each mitotic cell was analyzed and scored using NIS-Elements D 3.0. Typically, 160 mitotic cells were analyzed. The data represent the mean ± SD of three independent experiments (**P < 0.01, Student’s t-test).

A BER deficiency is expected to cause an accumulation of BER reaction intermediates, such as double-strand breaks (DSBs), which might eventually cause irreversible cell growth arrest or cell death by triggering apoptosis^34^. To determine whether the R137Q mutation can lead to an increase in DSBs, we examined proteins that are highly correlated with DSBs. Firstly, phosphorylation levels of histone H2AX were assessed via Western blot. As shown in Fig. 5a, H2AX phosphorylation was significantly increased in samples from an R137Q background. Secondly, to further verify this result, we counted the cells that had one or more γH2AX foci. We found that the percentage of cells with γH2AX foci was much higher in R137Q MEFs than it was in WT/R137Q MEFs and WT MEFs (Fig. 5b). Finally, as 53BP1 is a marker specifically recruited to DSBs during DNA damages, we performed a 53BP1 foci assay to confirm the occurrence of DSB. This assay showed that 53BP1 foci were significantly increased in the R137Q samples (Fig. 5c). Thus, these data collectively show that Pol β R137Q mouse embryos accumulate more DSBs than are found in WT embryos.

Because DSBs are considered to be critical primary lesions in the formation of chromosomal aberrations, we hypothesized that the DSB accumulation induced by the R137Q variant would further cause chromosomal aberrations. Metaphase spreads were analyzed to evaluate the difference between WT and R137Q MEFs. Giemsa staining showed that under normal conditions, R137Q MEFs possessed approximately 3-fold more single chromatid breaks than were observed in WT MEFs (Fig. 5d). This result indicates that increased levels of genomic instability are present in primary R137Q MEFs.

### R137Q MEFs are more sensitive to DNA damaging agents

**Figure 6:**
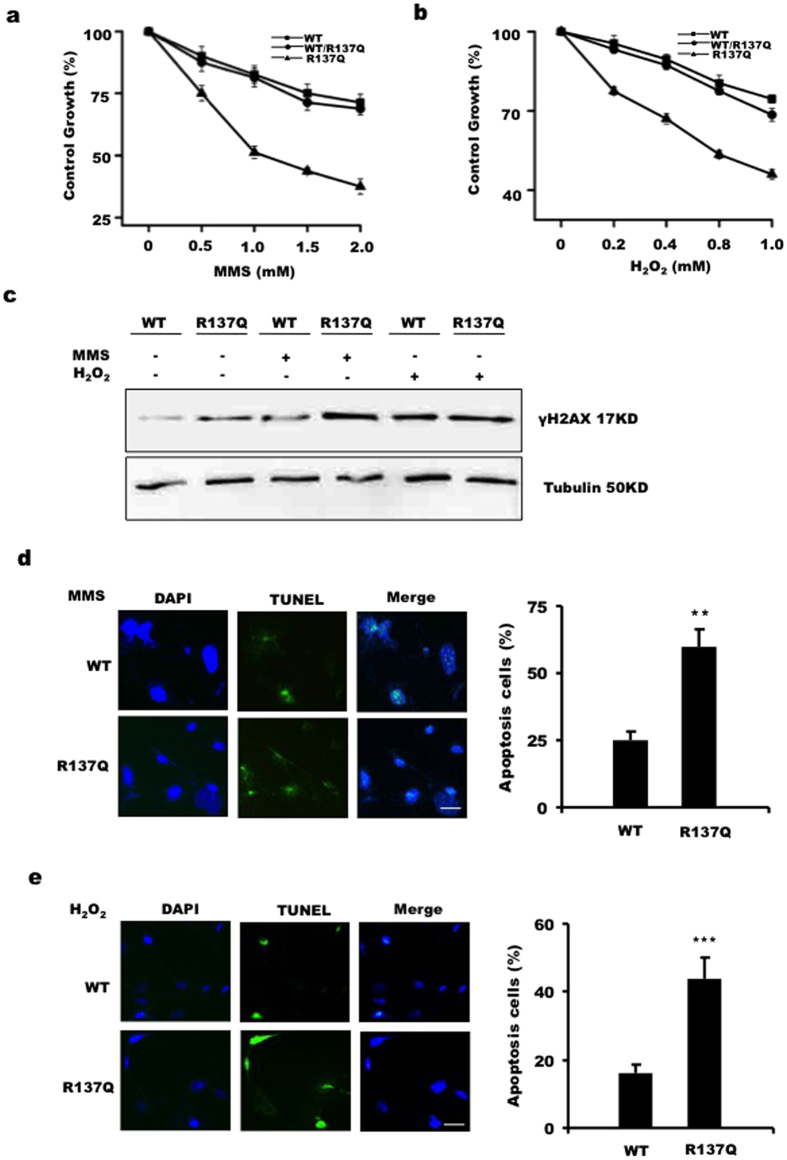
R137Q is more sensitive to MMS and H_2_O_2_. (**a,b**) Cells (1 × 10^5^ per well) were plated in triplicate in six-well plates. Cells were treated or untreated with different doses of MMS or H_2_O_2_ for 2 hours. After treatment, the cells were washed with PBS and cultured in a fresh media for 3 days. The number of viable cells in every well was determined following the trypsinization of the cells and counting with a cell counter (CountStar IC1000). The control growth ratio was calculated based on the treated/untreated cell numbers. The data represent the mean ± SD from triplicate wells. Three independent experiments were performed. (**c**) After treatment with MMS (1.5 mM) or H_2_O_2_ (0.8 mM), WT, WT/R137Q and R137Q MEFs were lysed to examine γH2AX protein level via Western blot. TUNEL assay results show that a stronger TUNEL-positive fluorescence signal was detected in R137Q MEFs after MMS (**d**) or H_2_O_2_ (**e**) treatment. Scale bar: 10 μm. Cells (3 × 10^3^) were analyzed, and the percentages of positive cells were calculated and plotted. Values are expressed as the mean ± SD of three independent experiments (**d**, **P < 0.01; **e**, ***P < 0.001, Student’s t-test).

Our data have shown that under normal conditions, Pol β R137Q impairs BER efficiency, leads to DSB accumulation, and causes chromosomal aberrations. All of these effects could directly or indirectly trigger apoptosis and cell death. Genomic DNA exposed to different types of endogenous and exogenous insults can rapidly suffer damage, which led us to expect that the cells carrying the R137Q mutant allele would be more sensitive to various DNA damaging agents. To test this hypothesis, WT, WT/R137Q and R137Q MEFs were treated with different doses of MMS or H_2_O_2_. We found that MMS and H_2_O_2_ consistently had a strong detrimental effects on cells (Fig. 6a,b). Following MMS or H_2_O_2_ treatment, the ratio of treated/untreated R137Q cells was significantly lower than that for WT or WT/R137Q cells. For further tests, MMS (1.5 mM) or H_2_O_2_ (0.8 mM) was chosen to treat MEFs. Western blot data show that after treatment, more γH2AX was detected in the R137Q group (Fig. 6c). A TUNEL assay was used to evaluate the apoptosis ratio after MMS or H_2_O_2_ treatment. Our results show that a stronger TUNEL-positive signal was detected from R137Q MEFs after MMS or H_2_O_2_ treatment (Fig. 6d,e). These data collectively indicate that the ability of a cell to respond to various DNA insults is significantly decreased by the R137Q mutation. In addition, a sensitivity assay was also performed in WT MEFs, R137Q MEFs and Pol β knockout MEFs (MEF88). The result show that MEF88 had the highest sensitivity to DNA damaging reagents (see Supplementary Fig. S2), which indicates that Pol β knockout results in a more impaired phenotype than that of the R137Q mutants.

## Discussion

Pol β plays a pivotal role in the BER pathway and is critical for maintaining the genomic DNA integrity. Arg137 is an important amino acid residue for the function of Pol β. Our previous study reported that the R137Q substitution decreased Pol β polymerase activity, disrupted its interaction with PCNA, and impaired its overall capacity to repair DNA base damage^20^. Here, we constructed Pol β R137Q knock-in mice and showed that homozygous knock-in mouse embryos are small, and have a high mortality rate (21%). Pol β R137Q mouse embryos exhibited decreased levels of cell proliferation and increased levels of apoptosis when compared with WT embryos. Primary MEFs isolated from the Pol β R137Q mice also showed reduced proliferation and a higher apoptosis ratio. These MEFs also accumulated BER reaction intermediate, such as DSBs, which may lead to apoptosis or genomic instability. Our results indicate that Pol β R137 is a key amino acid site, and that its mutation can severely impair the BER process, enhance apoptosis, and delay mouse embryo development.

Single nucleotide polymorphisms occur widely in DNA repair genes. For DNA Pol β, several non-synonymous single nucleotide substitutions have been identified (Q8R, R137Q, P242R and Y265C). These polymorphisms might result in biochemical alternations, BER deficiencies, and a predisposition to cancers^20^,^27^,^35^. Arginine 137 for example, an important residue for the function of Pol β, is located in helix 7 of the Pol β protein and forms hydrogen bonds with other adjacent amino acid residues. The amino acid substitution of Arg with Gln results in a net positive charge loss, which further disrupts the formation of hydrogen bonds, and impairs the protein’s polymerase activity. Our previous results from *in vitro* experiments suggest that R137Q’s contribution to the onset and development of cancers is due to its reduced polymerase activity, impaired BER efficiency, and increased sensitivity to DNA-damaging reagents^20^. However, as *in vitro* data can only partially explain R137Q’s biochemical functions, the exact physiological function of the mutation *in vivo* still needed further investigation. By using the Pol β R137Q knock-in mouse model we constructed, we found that the R137Q variant resulted in delayed embryo development due to an impaired DNA repair pathway.

Pol β contains two important domains, a dRP lyase domain and a polymerase domain^16^. Mice deficient for either of these two domains die after birth. The data from several knock-in mouse models have shown that the different site mutations could contribute differently to Pol β’s function. Y265C knock-in mice are born at normal Mendelian ratios but are smaller than normal, and 60% die within a few hours after birth^27^. In our study, we found that the litter size of R137Q knock-in mice was significantly smaller than that of WT mice. To explore the underlying mechanisms of these differences, we dissected the pregnant females and found that the R137Q embryos were small, and had a mortality rate of 21%. This phenotype is partly consistent with that of Y265C knock-in mice. Because Y265 and R137 are located at the same polymerase domain, Y265C and R137Q substitutions both significantly impair Pol β polymerase activity. However, the decreases in the activity ratios are different, which may lead to the small differences between the knock-in mouse phenotypes. The high embryonic mortality rate of R137Q mice (21%) shows that this mutation impairs embryo development at an early stage. This result also indicates that Arginine 137 is an important amino acid site, and its mutation would dramatically impairs Pol β function.

Living cells are constantly exposed to endogenous and exogenous insults, such as reactive oxygen, ionizing radiation and alkylating agents, which may cause DNA lesions (e.g., *O*^6^meG, 3meA and 7meG lesions)^36^. Therefore, cells have evolved a variety of repair mechanisms to fix these damages. Defects in DNA repair systems can promote the accumulation of secondary DNA lesions, such as single-strand breaks (SSBs). Then, during replication, collapsing replication forks leave the DNA backbone broken at the SSBs, which can be often converted to DSBs. DSBs are the most serious and lethal types of DNA damages and can induce apoptosis by activating exogenous death-receptor pathways or endogenous mitochondrial pathways^37^. A large amount of evidence shows that cell lines with defective DNA repair mechanisms are hypersensitive to DNA damage reagents and have strong apoptotic responses^38^. Here, our results show that Pol β R137Q knock-in mouse embryos exhibit the increased levels of double-strand breakages and chromosomal aberrations, indicating that their genomic DNA is partly damaged. Actually, the integrity of the genome is at a greater risk during embryo development because, cell proliferation occurs more rapidly during this period, DNA replication rates are high, and the cell cycle is much shorter in embryonic cells than it is in adult cells. Indeed, DNA repair at these early stages is of great significance for an organism’s proper development^39^. Many animals lacking DNA repair enzymes are not viable and suffer preimplantation death. Moreover, some small deficiencies in mammalian DNA repair systems could also lead to increased rates of birth defects, cancer, and reduced lifespans. This indicates that, the more important a DNA repair component is, the more serious the results will be if an individual lacks the component.

Previous studies show that Pol β is a key enzyme in the DNA repair systems, and that the knockout of Pol β results in mouse embryonic lethality. Our results suggest that the single-site mutation of Pol β (R137Q) could cause embryos to develop abnormally. However, we did not find that all embryos with this mutation were small or dead, which indicates that the R137Q mutation possesses a milder phenotype compared with that of Pol β knockout. This result may be due to the following two reasons. First, the R137Q mutation doesn’t completely disrupt the function of Pol β, it only decreases the enzyme’s polymerase activity and BER efficiency. Second, the apoptotic pathway is only activated in those cells with DNA that is damaged and not successfully repaired. Additionally, our MMS and H_2_O_2_ stimulation experiments show that R137Q MEFs are more sensitive to these DNA-damaging reagents and are more likely to undergo cell apoptosis than are WT MEFs. This result indicates that even if Pol β R137Q knock-in mice are born healthy, they will not be as healthy as WT mice throughout their lifetime. Once the knock-in mice are exposed to a certain amount of endogenous and exogenous DNA-damaging reagents, there would likely be an abundance of DNA lesions that could not be effectively repaired by their defective DNA repair systems. Therefore, R137Q knock-in mice might be more susceptible to developing cancer or other diseases, which needs further confirmation in future studies.

Summarily, our *in vivo* data support the hypothesis that Pol β is a key enzyme in the BER process, and that the R137Q mutation is an important polymorphism that severely impairs the function of Pol β. Our findings further suggest that BER is essential for maintaining the integrity of the genome during embryo development.

## Materials and Methods

### Pol β R137Q knock-in mice construction and breeding

We cloned a 6.7 kb fragment of mouse genomic DNA containing the appropriate segment of the Pol β gene and inserted the neomycin phosphotransferase gene (neo) between the two arms in the targeting vector. The TK gene was inserted downstream from the 5′ arm sequence to permit negative selection. A site-directed mutagenesis kit was used to change the nucleotides encoding Arg 137 to Gln 137. Then, the sequence-verified construct was electroporated into hybrid (129/Sv-C57BL/6) ES cells and selected clones that were neomycin resistant. Long PCR was used to screen the positive clones. The primers for the 5′ARM PCR were 5′AGGTGTGTACAATGTTGACTTGG3′ and 5′AGGTGTGTACAATGTTGACTTGG3′. The primers for the 3′ARM PCR were 5′TCTGCACGAGACTAGTGAGACGTGCTA3′ and 5′TGGGACAGAACATTCCTGTAGAGGTA3′. Two independent ES clones carrying the desired mutation were injected into C57BL/6 blastocysts. The chimeras were bred with actin-Flpe-C57BL/6 transgenic mice to screen for offspring carrying the germline-transmitted allele with the neomycin selection cassette removed. Many of the resulting newborns were *pol β*^+/q^ heterozygous. Mice carrying a copy of R137Q Pol β were bred to produce the first generation (F1) knock-in mice. Pol β cDNA was amplified via PCR using the primers 5′AGCAAGCAGCTACAATGCAA3′ and 5′AGGTGTGTACAATGTTGACTTGG3′ and sequenced to confirm the presence of the mutation encoding the R137Q variant. The experimental procedures and animal care protocols for this study were approved by the Institutional Animal Care and Use Committee of Nanjing Normal University, and all experiments were carried out in accordance with the approved guidelines.

### Reagents and antibodies

All primers and DNA substrates used in this paper were synthesized by GenScript Inc. using polyacrylamide gel electrophoresis (PAGE) purification. Four deoxynucleotide triphosphates (dNTPs) were purchased from New England Biolabs (N0446S). [α-^32^P]-dCTP (NEG513H, 250 μCi) was purchased from PerkinElmer. The γH2AX antibody (ab2893) and Pol β antibody (ab26343) were purchased from Abcam. The Ki67 antibody (sc-15402), tubulin antibody (sc-23950), and 53BP1 antibody (sc-22760) were purchased from Santa Cruz Biotechnology. The actin antibody (p-30002) was purchased from Abmart, and the AlexaFluor^®^594 donkey (A21207) was from Invitrogen.

### MEFs, cell treatments, and growth analysis

Homozygous mutant (R137Q), heterozygous mutant (WT/R137Q) and WT mouse embryonic fibroblast (MEFs) were derived from samples collected at embryonic day 16.5 and were cultured in DMEM containing 10% (vol/vol) FBS and 1% penicillin-streptomycin. To determine their sensitivity to the alkylating agent methyl-methanesulfonate (MMS) or H_2_O_2_, cells (1 × 10^5^ per well) were plated in triplicate in six-well plates in 2 mL DMEM containing 10% (vol/vol) FBS and 1% penicillin-streptomycin. The next day, cells were treated with MMS or H_2_O_2_ for 2 h. After treatment, cells were washed with PBS and cultured in fresh media for 3 days. Growth curves were determined by counting trypsinized cells with a cell counter (CountStar IC1000).

### Analysis of embryonic tissue and MEF cell apoptosis

WT and R137Q embryo tissue samples were fixed in a 10% buffered formalin solution and embedded in paraffin, and the paraffin sections were prepared for TUNEL analysis. MEFs (1 × 10^5^) were cultured on acid-cleaned coverslips. After growing under normal growth conditions (37 °C, 5% CO_2_) for 24 h, cells were fixed in 4% paraformaldehyde at room temperature for 30 min and treated with 0.2% Triton X-100 for 10 min. We used TUNEL kits (KGA7032, KGA7034) to detect apoptosis in tissue samples and MEFs.

### Western blots

Cell lysates were prepared according to standard protocols. The protein concentration was measured using the Bradford method. Equal amounts of protein were loaded, resolved on 10% SDS-PAGE, transferred onto a PVDF membrane (0.45 μm, KGP114), and incubated overnight at 4 °C with primary antibodies to the following compounds: tubulin (1:1,000), γH2AX (1:1,000), actin (1:1,000), Pol β (1:500). After extensive washing in PBS containing 0.1% Tween-20, the membrane was incubated with a 1:5,000 dilution of HRP-conjugated anti-mouse or anti-rabbit secondary antibody, for 1 h at room temperature. The membrane was then washed and developed via ECL (Tanno 4500).

### Immunofluorescence

Cells were cultured in six-well plates containing acid-cleaned coverslips and incubated under normal growth conditions (37 °C, 5% CO_2_) overnight. The coverslips were then washed with PBS, fixed with 4% formaldehyde in PBS for 30 min, and then washed again with PBS. Triton X-100 (0.2%) was added for 15 min to permeabilize the cells. Coverslips were blocked with 3% BSA for 30 min at RT and then incubated with a primary antibody. The coverslips were washed and then incubated with a FITC-conjugated secondary antibody, followed by washing with PBS and staining with DAPI. The mounted coverslips were viewed with a Zeiss Axioscope and images were captured with a charge-coupled device camera.

### BER assays

The SP-BER and LP-BER activities of Pol β were assayed using a synthetic DNA duplex (41 nt) carrying a U and F in the middle region. Complete repair reactions were carried out in 20 μl of a reaction buffer [40 mM HEPES–KOH (pH 7.8), 70 mM KCl, 7 mM MgCl_2_, 1 mM dithiothreitol, 0.5 mM EDTA, 2 mM ATP, 50 μM each of dATP, dTTP and dGTP, and 8 μM 2 μCi ^32^P-dCTP]. For the short-patch BER and long-patch BER assays, whole-tissue and cell extracts were incubated with the SP-BER substrate Pol β-U and the LP-BER substrate Pol β-F. Reactions (30 min, 37 °C) were then stopped by adding an equal volume of the gel-loading buffer, and visualized via autoradiography.

### Metaphase spread preparation and analysis

Cells were harvested following colcemid treatment (0.1 μM, 5 h), treated with a hypotonic solution (75 mM KCl), fixed with Carnoy’s solution, and mounted on a slide, which was pre-cooled at 4 °C for 2 h. Then, cells were stained with a Giemsa solution for 15 min. After staining, slides were examined under a microscope for mitotic cells, and the chromosome number of each mitotic cell was analyzed and scored using NIS-Elements D 3.0, with approximately 160 mitotic cells typically being analyzed.

### Statistical analysis

All data are expressed as the mean ± SD. Statistical significance was determined using a student t-test or analysis of variance (ANOVA) in the case of comparisons among more than two groups. P values less than 0.05 were considered significant.

## Additional Information

**How to cite this article**: Pan, F. *et al*. Mutation of DNA Polymerase β R137Q Results in Retarded Embryo Development Due to Impaired DNA Base Excision Repair in Mice. *Sci. Rep*. **6**, 28614; doi: 10.1038/srep28614 (2016).

## Supplementary Material

Supplementary Information
