# Supplementary material for: Mutation of DNA Polymerase β R137Q Results in Retarded Embryo Development Due to Impaired DNA Base Excision Repair in Mice
Source: Sci Rep. 2016 Jun 30;6:28614. doi: 10.1038/srep28614 (PMC4928080; doi:10.1038/srep28614)
Supplement: Supplementary Information [file srep28614-s1.pdf]

Mutation of DNA Polymerase β R137Q Results in Retarded Embryo Development Due to Impaired DNA Base Excision Repair in Mice

Feiyan Pan[1,a], Jing Zhao[1,a], Ting Zhou[1,a], Zhihui Kuang[2], Huifang Dai[3], Huan Wu[1], Hongfang Sun[1], Xiaolong Zhou[1], Xuping Wu[2], Zhigang Hu[1], Lingfeng He[1], Binghui Shen[3], Zhigang Guo[1]*

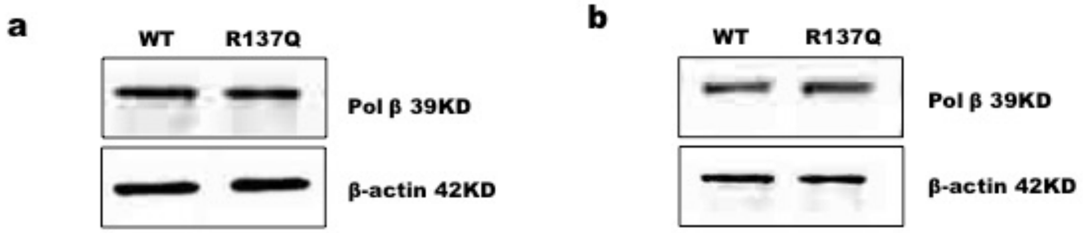

Supplementary Figure. S1 The protein levels of Pol β in tissues and cell lysate from WT and R137Q. Pol β protein levels were examined by Western blot. Tubulin was immunoblotted as a loading control. **(a)** Tissue lysate. **(b)** Cell lysate.

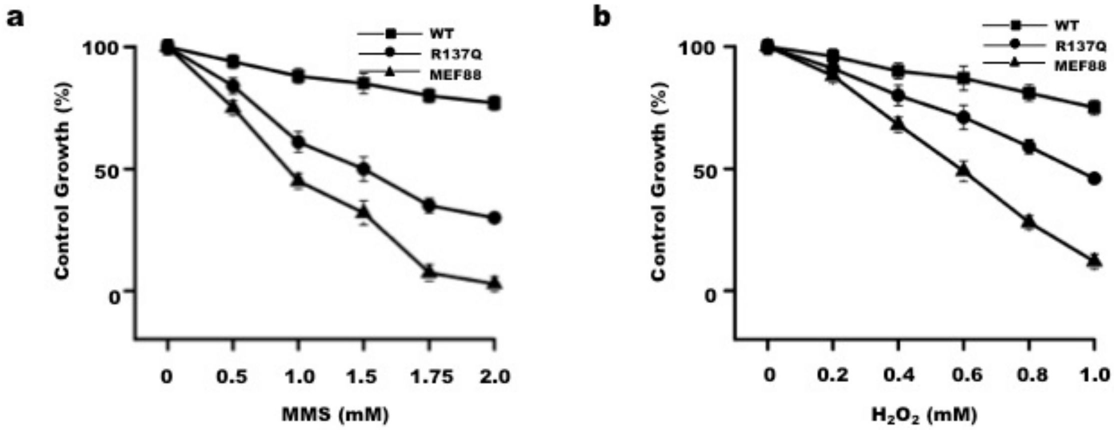

Supplementary Figure. S2 Sensitivity assay in WT, R137Q and Pol β knockout MEFs. 1×10^5 cells per well were plated in triplicate in six well plates, cells were treated or untreated with different dose of MMS or H_2O_2 for 2 hours. After treatment, the cells were washed with PBS and cultured in a fresh media for 3 days. The number of viable cells in every well was determined by trypsinized cells and counting with a cell counter (CountStar IC1000). The control growth ratio was calculated by treated/untreated cell numbers. Pol β knockout MEFs, MEF88. Data represents mean±SD from triplicate wells. The experiments were repeated three times.
